# Salt loading decreases urinary excretion and increases intracellular accumulation of uromodulin in stroke-prone spontaneously hypertensive rats

**DOI:** 10.1042/CS20211017

**Published:** 2021-12-17

**Authors:** Sheon Mary, Philipp Boder, Giacomo Rossitto, Lesley Graham, Kayley Scott, Arun Flynn, David Kipgen, Delyth Graham, Christian Delles

**Affiliations:** 1 Institute of Cardiovascular and Medical Sciences, University of Glasgow, Glasgow, Scotland, U.K.; 2 Department of Medicine, University of Padua, Padua, Italy; 3 Department of Pathology, Queen Elizabeth University Hospital, Glasgow, Scotland, U.K.

**Keywords:** blood pressure, renal physiology, Salt, salt-sensitive hypertension, Tamm Horsfall protein

## Abstract

Uromodulin (UMOD) is the most abundant renal protein secreted into urine by the thick ascending limb (TAL) epithelial cells of the loop of Henle. Genetic studies have demonstrated an association between UMOD risk variants and hypertension. We aimed to dissect the role of dietary salt in renal UMOD excretion in normotension and chronic hypertension. Normotensive Wistar–Kyoto rats (WKY) and stroke-prone spontaneously hypertensive rats (SHRSP) (*n*=8/sex/strain) were maintained on 1% NaCl for 3 weeks. A subset of salt-loaded SHRSP was treated with nifedipine. Salt-loading in SHRSP increased blood pressure (ΔSBP 35 ± 5 mmHg, *P*<0.0001) and kidney injury markers such as kidney injury marker-1 (KIM-1; fold change, FC 3.4; *P*=0.003), neutrophil gelatinase-associated lipocalin (NGAL; FC, 2.0; *P*=0.012) and proteinuria. After salt-loading there was a reduction in urinary UMOD excretion in WKY and SHRSP by 26 and 55% respectively, compared with baseline. Nifedipine treatment reduced blood pressure (BP) in SHRSP, however, did not prevent salt-induced reduction in urinary UMOD excretion. In all experiments, changes in urinary UMOD excretion were dissociated from kidney UMOD protein and mRNA levels. Colocalization and *ex-vivo* studies showed that salt-loading increased intracellular UMOD retention in both WKY and SHRSP. Our study provides novel insights into the interplay among salt, UMOD, and BP. The role of UMOD as a cardiovascular risk marker deserves mechanistic reappraisal and further investigations based on our findings.

## Introduction

Uromodulin (UMOD) is the most abundant protein in the urine of healthy individuals. It is secreted by the epithelial cells of the thick ascending limb (TAL) of the loop of Henle in the kidney. Genome-wide association studies have identified UMOD variants to be associated with renal function, chronic kidney disease and hypertension [1,2]. However, the biological role of UMOD in the pathogenesis of hypertension and kidney diseases is still elusive [3].

There is growing evidence that UMOD is critically involved in sodium homeostasis and thus in blood pressure (BP) regulation [4,5]. Overexpression of UMOD in transgenic mice (FVB/N) lead to renal injury and salt-sensitive hypertension owing to increased activation of NKCC2 [4]. Contrary to the overexpression model, young UMOD knock-out mice were found to be resistant to salt-induced BP rise [5]. Matafora et al. have shown that UMOD secretion is greater in salt-sensitive and resistant hypertensive patients compared with healthy individuals and that the levels of UMOD correlate inversely with sodium excretion [6]. In humans, both serum and urinary UMOD excretion have been inversely associated with cardiovascular disease (CVD) and risk of progressive kidney disease [7–9]. However, the mechanisms underlying this association remain incompletely understood. If taken out of due context, the inverse nature of the association would appear paradoxical, based on findings by Graham et al. and Trudu et al. [4,5].

To date, mouse models such as UMOD knock-out, transgenic overexpression model and hepsin knock-out, all lacking a background of hypertension, were used to dissect the role of UMOD in the pathogenesis of (predominantly salt-sensitive) hypertension [4,5,10]. Retrospective analysis of the DASH-Sodium trial showed that higher urine UMOD levels were not associated with an increase in BP in response to an increase in salt intake [11]. Recently, an interventional study in a Chinese cohort showed that high salt intake reduced significantly both plasma and urinary UMOD levels [12].

Our present study aimed to offer a novel perspective for the interpretation of the human association studies above. As such we explored a reverse question, i.e. whether salt (and/or hypertension) could modulate UMOD from expression to excretion, and if so, whether there is a difference between normotensive and chronic hypertensive conditions. In our study, we used the stroke-prone spontaneously hypertensive rat (SHRSP), a unique model of polygenic hypertension and CVDs. According to the Rat Phenome Project the SHRSP exhibits the highest BP among 179 rat strains [13]. They are well-studied model for chronic hypertension [14], stroke [15], vascular dysfunction [16], and renal damage [17]. Genetic predisposition to develop renal damage has been observed in SHRSP [17]. Salt supplementation in SHRSP increases this severity of hypertension and magnifies the renal damage [17].

Specifically, we aimed to investigate whether: (i) salt affects UMOD excretion in TAL; (ii) dietary salt has the same effect on UMOD excretion in normotensive and hypertensive animals; (iii) the effect of salt on UMOD excretion is dependent on BP. To study the latter, we treated salt-loaded SHRSP with calcium channel blocker nifedipine which has previously been shown to alleviate salt-induced hypertension in SHRSP [18,19].

## Materials and methods

### Animals and experimental design

All procedures were performed in accordance with Home Office regulation and with the United Kingdom Animals Scientific Procedures Act 1986 (PPL No. 70/9021) and ARRIVE Guidelines and were approved by the institutional ethics review committee and performed at University of Glasgow. Animals were housed under controlled environmental temperatures (21 ± 3°C) and lighting (12-h light–dark cycles) and maintained on standard rat diet (rat no. 1 maintenance diet; Special Diet Services, Grangemouth, United Kingdom) and were provided tap water *ad libitum*. In the present study, we used both male and female SHRSP (*n*=54) and Wistar–Kyoto rat (WKY) (*n*=32) at 11 weeks of age (hereafter: ‘baseline’). At 12 weeks of age, littermates were randomized into groups of *n*=8/sex/group to normal drinking water (normal salt, NS) and 1% NaCl (hereafter: ‘1% salt’) water (high salt, HS) for 3 weeks. A subset of SHRSP on HS (*n*=8/sex) was treated with the calcium channel blocker nifedipine (Sigma, Dorset, U.K.), provided daily for 3 weeks at 10 mg/kg/day in 1 ml of baby food (Heinz Custard) and in parallel, at 15 mg/kg/day in drinking water as previously established [20]. To maintain consistency among groups, the baby food was fed to all rats (with/without nifedipine). Systolic blood pressure (SBP) was monitored weekly by tail-cuff plethysmography [21], in an operator-blinded fashion.

### Sample collection

#### Urine collection

A fixed amount of water (250 ml) was given, and daily intake recorded; food was available *ad libitum* over the 24 h. Animals were individually housed in metabolic cages at baseline and once every experimental week to estimate 24-h urine output. Urine samples were collected during these times. Animals were acclimatized for 2 h, 3 days before the first measurement. Twenty-four hour-urine samples were aliquoted and stored at −80°C.

#### Blood collection

Heparinized blood was collected by tail vein puncture (baseline and first 2 weeks of the experiment) under anesthesia (isoflurane). At the end of the 3-week-study, heparinized and EDTA blood was collected by cardiac puncture and rats were killed by exsanguination under terminal general isoflurane anesthesia. Biochemical plasma and urinary analyses for electrolyte, albumin and creatinine concentration were performed using Roche Cobas C311 Analyzer and commercially available rodent kits (Roche, Sussex, U.K.).

#### Tissue collection

Kidneys were dissected into two halves. One part was formalin fixed and paraffin-embedded and the other was snap-frozen in liquid nitrogen and stored at −80°C until use.

### UMOD quantitation by ELISA

Urinary and total kidney (lysate) UMOD concentration was quantified using ELISA kit (Abcam, Cambridge, U.K.) as per the manufacturer’s guidelines for SimpleStep ELISA. The range of detection for UMOD standards was 625–40000 pg/ml. Urine samples were diluted accordingly. All samples were quantified on the same day to minimize variation. Plates were read at 450 nm in a microplate reader (Victor Multilabel Plate Reader X3, Perkin Elmer Inc., Waltham, US.A.). For assessment of kidney UMOD protein, the kidney was chopped into smaller pieces and washed in PBS before homogenization in cell extraction buffer provided with the ELISA kit and using TissueLyser II (Qiagen, Manchester, UK.). The lysate protein quantification was determined by Bradford assay (QuickStart Bradford protein assay, Bio-Rad Laboratories Ltd, Hertfordshire, UK.). and equal amount of protein was loaded for ELISA. To minimize variation all sample protein extraction was performed on the same day. During the experiments, the analyst was blinded to sample details.

### Histology and immunofluorescence analysis

Paraffin-embedded kidneys were sectioned (2 µm) for histological staining and immunofluorescence. *n*=8/group (4/sex) kidneys were evaluated and scored in a blinded fashion. Periodic acid–Schiff stain was used to assess morphological differences. For immunofluorescence, kidney sections were placed in hot citrate buffer (pH 6.0 at 95°C) for antigen retrieval. This was followed by a blocking step with 1× Carbo-Free™ blocking solution (Vector Laboratories, California, U.S.A.). Then sections were incubated with goat anti-calnexin (1:100; Abcam, Cambridge, U.K.). After washing steps, the sections were incubated in donkey anti-goat IgG (H+L) cross-adsorbed secondary antibody, Alexa Fluor 546 (1:500; Thermo Fisher Scientific, Paisley, U.K.). These sections then underwent a secondary sequential antibody incubation, with rabbit anti-UMOD (1:2000; Abcam, Cambridge, U.K.) and goat anti-rabbit IgG (H+L) highly cross-adsorbed secondary antibody, Alexa Fluor 647 (1:500; Thermo Fisher Scientific, Paisley, U.K.). All antibodies were diluted in phosphate-buffered saline supplemented with 0.1% Tween-20 and incubations occurred for 1 h at room temperature. Sections were mounted in ProLong Gold antifade mounting medium (Thermo Fisher Scientific, Paisley, U.K.) and images were acquired with Zeiss Observer Z1 SpinningDisk confocal microscope (Carl Zeiss, Oberkochen, Germany) equipped with a Yokogawa filter wheel and spinning disc unit, a Photometrics Evolve EM-CCD camera, operating with Zen Blue software (Carl Zeiss, Oberkochen, Germany). Images were taken with objectives: Plan-Neofluar 25×/0.8NA and C-Apochromat 40×/1.2NA water immersion lens. Images were processed using Zen Blue Lite software and analyzed with ImageJ (http://imagej.nih.gov/ij/). Staining, imaging and scoring were performed by different analysts in a blinded fashion. An open source ImageJ plugin EzColocalization was used to quantify colocalization of UMOD and calnexin [22].

### Quantitative real-time PCR

Isolation of total RNA from kidney was performed using RNeasy mini spin kit (Qiagen, Manchester, U.K.) and the subsequent reverse transcription with High Capacity RNA to cDNA kit (Thermo Fisher Scientific, Paisley, U.K.). Gene expression assay was performed using rat-specific TaqMan probes (Thermo Fisher Scientific, Paisley, U.K.): *Umod*, *Havcr1* (*Kim-1*), *Lcn2* (*NGal*), and *Actb* and TaqMan Fast Advanced Master Mix. The expression levels were normalized to the housekeeping gene and presented as δ*C*_t_ values (inversely proportional to gene expression level).

### Isolation of medullary TAL tubules from rat kidneys

Isolation of medullary TAL tubules was performed as previously described [22]. Kidney of additional WKY and SHRSP of the age 15 ± 1 weeks were used for tubule isolation. Briefly, the kidney was cut along the corticopapillary axis and the inner stripe of the outer medulla was dissected and minced in 0.1% (w/v) collagenase solution prepared in Hanks’ Balanced Saline Solution (HBSS). This solution was incubated for 10 min at 37°C. The cell suspension was sedimented on ice and mixed with HBSS containing 2% (w/v) BSA, and the crude suspension of tubules was collected. Tubule suspension was spun at low speed (1000 rpm) for 10 min and resuspended in HBSS. The resuspension was passed over a 52-µm nylon mesh membrane (Fisher Scientific, Loughborough, U.K.). The tubules collected on the mesh were washed with HBSS and centrifuged for 5 min at 500 rpm. The supernatant was aspirated, and the cells were resuspended with DMEM (with sodium pyruvate and glutamine). The tubule suspension was then aliquoted into tubes for incubation in DMEM with either (i) nifedipine (10 mM) or (ii) salt-stress experiment (154 mM in addition to the pre-existing sodium in media). The tubules were incubated for 4 h at 37°C in a rotating incubator. After incubation, the tubule suspension was centrifuged at 1000 rpm for 10 min, and the supernatant was collected and stored at −80°C until further use. The tubule pellet was lysed with either (i) Qiazol for RNA and protein isolation (nifedipine incubation) or (ii) Mem-PER™ plus membrane protein extraction kit (Thermo Fisher Scientific, Paisley, U.K.) for membrane and cytosolic protein extraction (salt-stress experiment). Due to the low yield of tubules per kidney dissection, the samples were pooled per strain and incubation was performed in triplicate per experiment. The results represent two experiments performed each with WKY (*n*=4) and SHRSP (*n*=6) kidneys.

### Western blot

Proteins were separated on 4–12% NuPAGE (Thermo Fisher Scientific, Paisley, U.K.). Semi-dry blotting (Power Blot, Thermo Fisher Scientific, Paisley, U.K.) was performed on low-fluorescence PVDF membrane (Merck, Dorset, U.K.). The amount of protein loaded was different for blot depending on optimization: 1–5 µg for urine UMOD, 50 µg for kidney lysate, and 10–20 µg for *ex-vivo* tubule lysate. Membranes were incubated with 5% BSA solution at room temperature, followed by overnight primary antibody incubation at 4°C. Primary antibody used are: anti-UMOD (1:1000, Abcam, Cambridge, U.K.), anti-UMOD (1:1000 R&D Systems, Abingdon, U.K.), anti-calnexin (1:1000; Abcam, Cambridge, U.K.), anti-calreticulin (1:1000, Abcam, Cambridge, U.K.), anti-hepsin (1:500, Abcam, Cambridge, U.K.), anti-flotillin (1:1000, Abcam, Cambridge, U.K.), and anti-β-actin (1:20000, Sigma–Aldrich, Dorset, U.K.). Secondary antibodies used are: anti-rabbit (1:10000, Alexa Flour 800, Thermo Fisher Scientific, Paisley, U.K.), anti-goat (1:10000, Alexa Flour 800, Themo Fisher Scientific, Paisley, U.K.), anti-sheep (1:10000, Alex Fluor 680, Thermo Fisher Scientific, Paisley, U.K.), and anti-mouse (1:10,000, Alexa Fluor 680, Thermo Fisher Scientific, Paisley, U.K.) were used for detection. Imaging was performed on Odyssey Clx and analysis on ImageStudio v5.0 (LI-COR Biotechnology, Cambridge, U.K.). In case of membrane and cytosol blots of *ex-vivo* tubule experiment Revert 700 total protein stain (LI-COR Biotechnology, Cambridge, U.K.) was used for normalization.

### Statistics

Statistical analysis was performed on GraphPad Prism version 8 or IBM SPSS version 25. Student’s *t* test or ANOVA or Welch test or Mann–Whitney test was performed as appropriate. The effect of two factors (i.e. strain and time or salt) was tested by two-way ANOVA (or mixed model). Figure legends denote the type of test used for analysis. All statistical tests were two-tailed and *P*-value <0.05 was considered significant. For ELISA analysis, four-parameter curve fit (4PL) without constraints was used to determine the curve fit for standard values in GraphPad Prism. In the case of qPCR, the *C*_t_ values obtained from QuantStudio software were manually used for calculation of δ*C*_t_ and fold change (FC) in Excel, and the graphs were created in GraphPad. For Western blot, β-actin was used for normalization and further sum of replicate methodology for normalization between blots [23].

## Results

### BP and renal morphological changes upon salt loading

**Figure 1:**
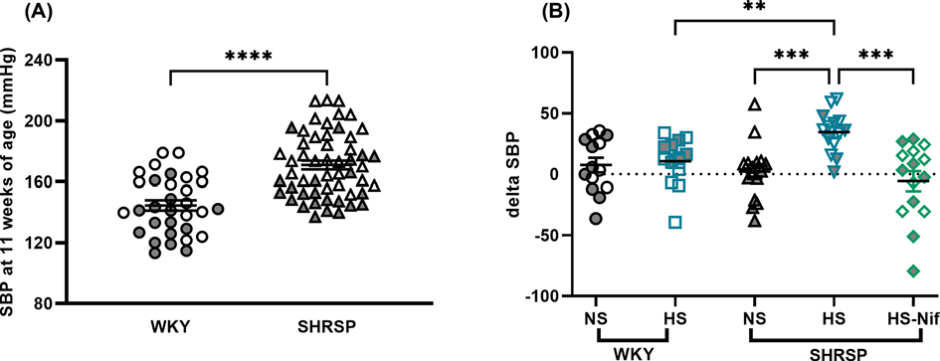
Salt-loading increases BP in SHRSP SBP was assessed by tail-cuff measurements. Each single dot represents an average of six to ten consecutive weekly SBP recordings. (**A**) shows the difference in SBP between 11-week-old WKY (*n*=32) and SHRSP (*n*=54). ****P<0.0001 (Welch’s *t* test). Sexual dimorphism in SBP was observed in SHRSP (female shaded gray). (**B**) shows differences in SBP (ΔSBP) between baseline (11-week-old) and after 3 weeks of salt loading. WKY were divided into two groups (*n*=16/group): normal salt (NS; no salt drinking water) and high salt (HS; 1% NaCl in drinking water). SHRSP were divided into three groups (*n*=16/group): NS, HS and high salt with nifedipine (HS-Nif). Nifedipine prevented the salt-induced increase in SBP in salt loaded SHRSP. **P<0.01, ***P<0.001 (Brown–Forsythe Welch ANOVA test). Symbols with gray fill indicate female. Bars indicate mean ± s.e.m.

In keeping with previous reports [24], 11-week-old control SHRSP had significantly higher systolic BP (ΔSBP, 20 ± 5 mmHg, *P*<0.0001) than age-matched WKY (Figure 1A). We also observed a sexual dimorphism in BP [25], with male SHRSP exhibiting higher BP than females (Figure 1A). SHRSP and WKY exhibited contrasting susceptibility to salt-induced hypertension on 1% salt loading, a gradual increase in SBP was observed only in SHRSP (Figure 1B and Supplementary Figure S1). In particular, 3 weeks of 1% salt loading raised SBP from baseline in SHRSP (ΔSBP 35 ± 5 mmHg, *P*<0.0001) but not in WKY (ΔSBP 11 ± 6 mmHg, *P*=0.081) (Figure 1B). To counteract the salt-induced rise in BP, we treated salt-loaded SHRSP with nifedipine; this lowered SBP (ΔSBP −6 ± 8 mmHg, *P*=0.491) (Figure 1B and Supplementary Figure S1).

We observed mild abnormalities in renal histology related to salt loading in the two strains. Scoring for glomerular ischemia and arterial intimal thickening showed no significant difference among groups (**S2**). Notably, the male salt-loaded SHRSP showed arteriolar hyalinosis associated with higher BP rise (**S2**). Proximal tubules showed no acute injury in any groups; however, the distal tubules of SHRSP, regardless of salt or treatment, showed mild flattening and loss of occasional tubular epithelial nuclei (Supplementary Figure S2). Overall, the 1% salt loading for 3 weeks in SHRSP did not induce severe histological kidney damage.

### Differences in renal adaption to salt stress between WKY and SHRSP

Renal parameters relevant to Na^+^ and water homeostasis in rats were measured at the end of the experimental protocol (baselines were comparable across groups; Suppementary Figure S3). Salt-loaded rats of both strains drank considerably more water; both 24-h urine output and 24-h urinary sodium excretion increased accordingly. Treatment with nifedipine at least partially reversed all these effects. Plasma Na^+^ remained similar and unaffected by treatments across groups. Notably, net balance estimations revealed retention of both water (*P*=0.046 vs control SHRSP; unmeasurable water losses for absolute balance calculation could not be assessed) and Na^+^ in salt-loaded SHRSP (*P*=0.002 vs the null hypothesis of even intake and output), which were entirely corrected by nifedipine (*P*<0.001 and *P*=0.011, respectively).

**Figure 2:**
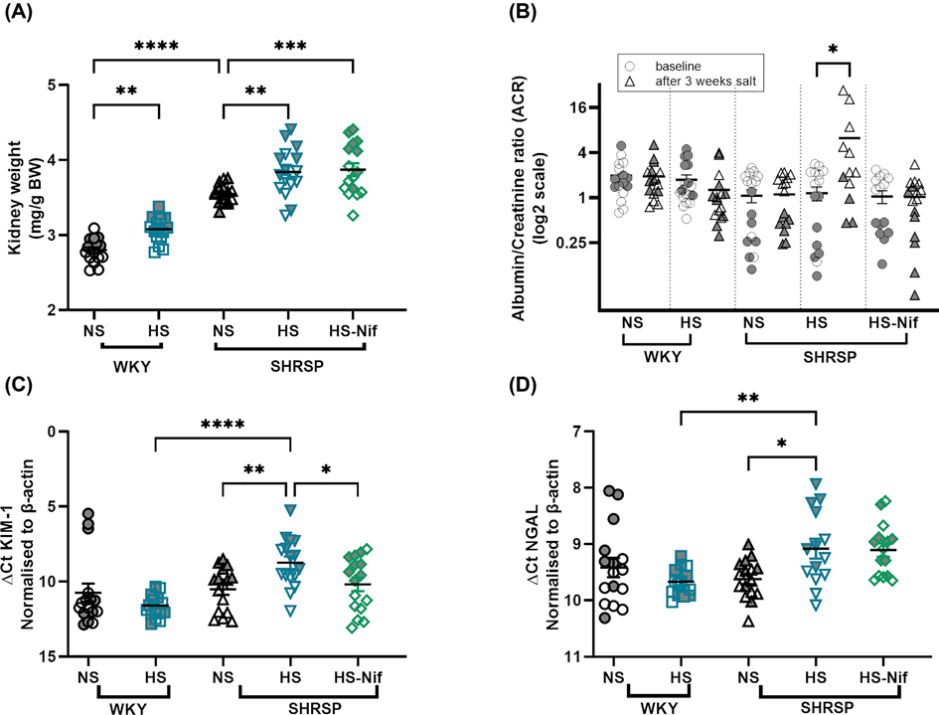
Salt-loading induced kidney injury markers in SHRSP (**A**) shows the significant difference in kidney weight among strains. Salt-loading significantly increased the kidney weight. **P<0.01, ***P<0.001, ****P<0.0001 (ANOVA with Bonferroni’s multiple comparison test). (**B**) shows the significant difference in ACR in SHRSP after salt-loading which is ameliorated with nifedipine treatment. *P<0.05 (Student’s *t* test). (**C,D**) represent mRNA expression of kidney injury marker-1 (KIM-1) and neutrophil gelatinase-associated lipocalin (NGAL), respectively. *P<0.05, **P<0.01, ****P<0.0001 (Brown–Forsythe Welch ANOVA test). NS: normal salt, HS: high salt (1% NaCl) and HS-Nif: high salt with nifedipine, *n*=16 per group. Symbols with gray fill indicate female. Bars indicate mean ± s.e.m.

Kidney weight was significantly different between strains, where SHRSP kidneys weighed more than WKY kidneys suggesting functional hypertrophy (Figure 2A). Salt loading led to a mild yet significant increase in kidney weight in both strains; this increase was unchanged with nifedipine treatment in salt-loaded SHRSP (Figure 2A). At baseline, albumin to creatinine ratio (ACR) in WKY and SHRSP was not significantly different; however, salt loading led to a marked elevation of ACR in SHRSP compared with baseline and all other groups. This elevation was abolished by nifedipine (Figure 2B).

We investigated early diagnostic molecular tubular injury markers such as kidney injury marker-1 (KIM-1; proximal tubule) and neutrophil gelatinase-associated lipocalin (NGAL; TAL and distal tubule). KIM-1 expression was elevated in salt-loaded SHRSP compared with animals on normal salt (FC, 3.4; *P*=0.003) (Figure 2C). Similarly, NGAL expression was also significantly elevated in salt-loaded SHRSP (FC, 2.0; *P*=0.012) compared with animals on normal salt (Figure 2D). Administration of nifedipine ameliorated the expression of KIM-1 (Figure 2C), however, NGAL expression remained higher in SHRSP on high salt (Figure 2D).

### Salt-loading decreases 24-h urinary UMOD excretion in both SHRSP and WKY

**Figure 3:**
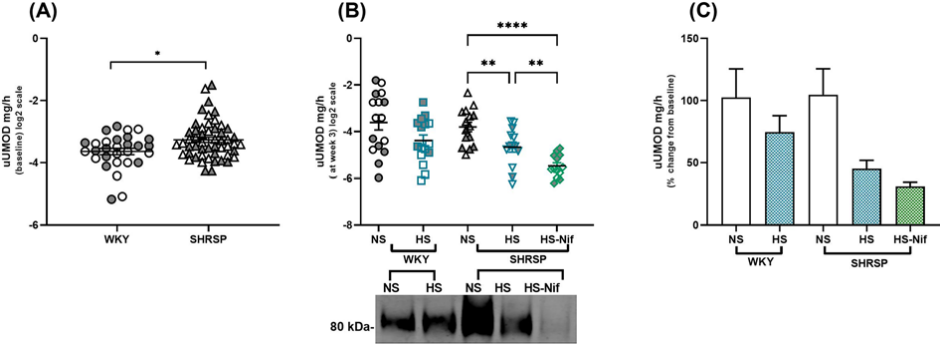
Urinary UMOD decreases with salt-loading in both WKY and SHRSP Twenty-four hour urine samples were collected from rats caged individually once a week in metabolic cages. Urinary UMOD (uUMOD) concentration in urine samples were quantified by ELISA and normalised to 24-h urine output. (**A**) represents the difference in baseline (11-week-old rats) 24-h uUMOD between WKY (*n*=32) and SHRSP (*n*=54). *P<0.05 (Mann–Whitney test). Salt loading decreased uUMOD excretion in urine in both strains, and nifedipine treatment did not change the salt-induced reduction of uUMOD. (**B**) compares the 24-h uUMOD (ELISA) between groups at week 3 of salt-loading. Western blot represents pool of samples from each group. **P<0.01, ****P<0.0001 (Brown–Forsythe Welch ANOVA test). (**C**) represents the percentage change of uUMOD from baseline after 3 weeks of salt-loading in each group. Bars indicate mean ± s.e.m; NS: normal salt, HS: high salt (1% NaCl) and HS-Nif: high salt with nifedipine, *n*=16 per group. Symbols with gray fill indicate female.

To study the effect of salt on urinary UMOD excretion, we first analyzed the concentration of UMOD in 24-h urine. At baseline, the urinary UMOD excretion rate in SHRSP was significantly higher than in age-matched WKY (Figure 3A). With salt loading, we observed a gradual decrease in urinary UMOD from week 1 through week 3 in WKY and SHRSP (Supplementary Figure S4). Compared with rats on normal salt, at week 3, there was a decrease in urinary UMOD excretion in both salt-loaded WKY (FC, 0.5; *P*=0.028) and SHRSP (FC, 0.6; *P*=0.018) (Figure 3B). By week 3, compared with baseline, urinary UMOD excretion was unchanged in rats exposed to normal salt but was reduced by 26and 55% in WKY and SHRSP exposed to high salt, respectively (Figure 3C). From renal histology, we could confirm that structural tubule damage in SHRSP was unlikely to be the cause of the decrease in urinary UMOD excretion, observed also in the functionally and structurally unaffected salt-loaded WKY.

Upon salt loading, but not on a normal salt diet and regardless of group or treatment, urinary UMOD excretion was inversely associated with absolute and fractional excretion of Na^+^ (Spearman ρ = −0.502, *P*=0.0005 and ρ = −0.368, *P*=0.016, respectively; Supplementary Figure S5). NKCC2 abundance (protein and mRNA) and phosphorylation in different groups showed no difference (Supplementary Figure S6).

We determined whether nifedipine influences the salt-induced decrease in urinary UMOD excretion. In salt-loaded SHRSP, nifedipine led to a further decrease in urinary UMOD excretion rate (FC, 0.5; *P*=0.004) compared with control animals (Figure 3B), and down to a total of 67% reduction in urinary UMOD excretion from baseline (Figure 3C). This suggests a salt-induced decrease in urinary UMOD excretion irrespective of strain and antihypertensive treatment.

### Urinary UMOD excretion does not reflect its mRNA and kidney protein level

**Figure 4:**
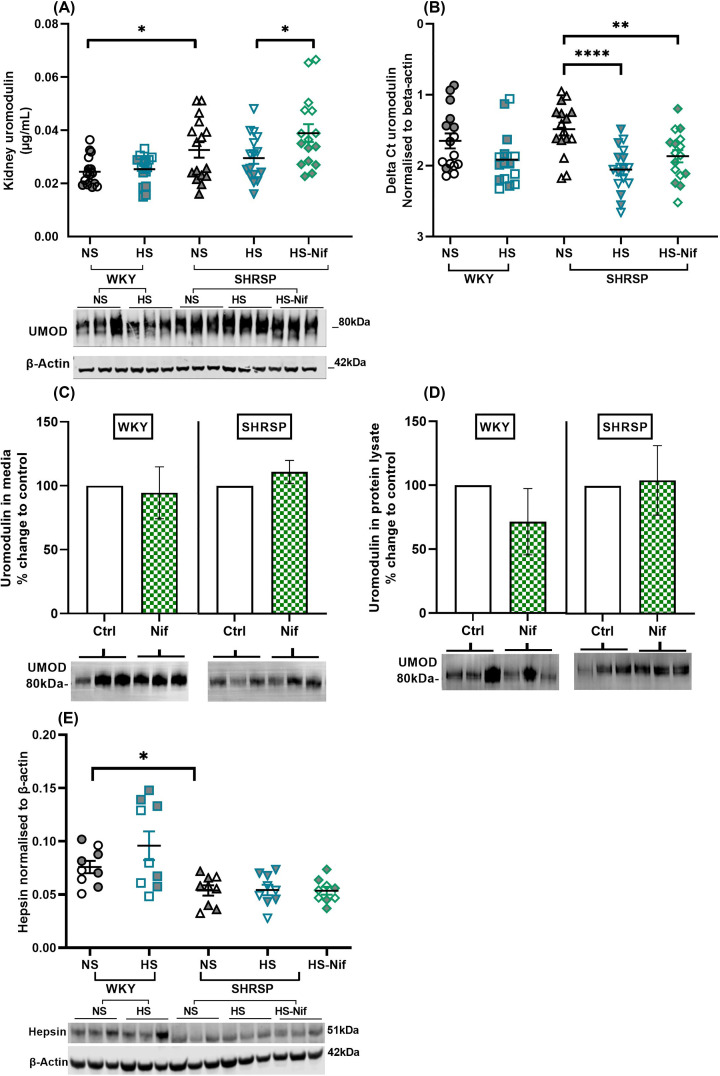
uUMOD excretion does not reflect its mRNA and total kidney protein level One-fourth of kidney (cut into sagittal plane and then into two halves) was used for RNA and total protein isolation (*n*=16 per group). (**A**) shows the significant difference in total kidney UMOD between strains and no significant difference with salt loading. Top: Graph represents results of UMOD ELISA normalised to total protein; Bottom: representative Western blot image of each group. (**B**) represents the comparison of δ*C*_t_ of *Umod* normalised to β-actin within strains. (**C**,**D**) represents the *ex-vivo* experiment, wherein, medullary tubules were isolated from kidneys of WKY and SHRSP (12 ± 3 weeks old). The tubules were incubated either with 10 mM nifedipine (Nif) for 6 h at 37°C. The results are representation of two experiments performed each with WKY (*n*=4) and SHRSP (*n*=6) rats. (C) shows no significant change in UMOD secretion in media from both WKY and SHRSP tubules when incubated with nifedipine. (D) shows no change in UMOD in tubule protein lysate in both strains after incubation with nifedipine. Western blots at the bottom of panels (A,B) are representative images. Ctrl = control. Full image of Western blot and analysis can be found in Supplementary Figure S8. (**E**) shows the strains (*n*=9 per group) significant difference in hepsin expression in kidney, which is unaffected with salt. Western blot is representative image of each group. *P<0.05, **P<0.01, ****P<0.0001 (Brown–Forsythe Welch ANOVA test). Bars indicate mean ± s.e.m. NS: normal salt, HS: high salt (1% NaCl) and HS-Nif: high salt with nifedipine. Symbols with gray fill indicate female.

Next, we investigated whether the salt-induced decrease in urinary UMOD excretion was at the transcriptional, translational or post-translational level. Similar to the strain differences observed for urinary UMOD excretion rate, the whole kidney protein levels of UMOD were also higher in SHRSP than WKY (Figure 4A). However, total kidney UMOD was not different after salt loading in both strains (Figure 4A). Notably, there was a slight decrease in UMOD mRNA levels in WKY and SHRSP with salt loading, which reached statistical significance only in SHRSP (relative FC compared with normal salt, 0.7; *P*<0.001; Figure 4B and Supplementary Figure S7). The inconsistency of trends for urinary excretion versus synthesis (mRNA) and total kidney UMOD suggests down-regulation of UMOD cellular secretion upon salt loading (see below). In nifedipine-treated, salt-loaded SHRSP, despite unaltered mRNA levels, UMOD kidney protein levels were increased by 32% (*P*=0.027) compared with animals on normal salt, (Figure 4A,B). This increased accumulation of total kidney UMOD could explain the lower urinary UMOD excretion (extracellular) in nifedipine treated salt-loaded SHRSP compared with salt-loaded SHRSP. Since WKY rats become hypotensive when treated with nifedipine [26,27], we did not give nifedipine to salt-loaded WKY.

To determine whether nifedipine had any direct effects on TAL tubules, we incubated isolated TAL tubules from WKY and SHRSP with nifedipine. Incubation with nifedipine did not change the secretion of UMOD into media (Figure 4C) and the concentration in cell lysate (Figure 4D) compared with control in both strains. Our data suggest that nifedipine is efficient in systemically reducing the BP under salt stress in SHRSP, while it does not have any direct effect on UMOD in TAL tubules under salt stress. In fact, L-type calcium channels are not reported to be present on TAL cells [28].

In light of the unaffected total kidney UMOD despite lower excretion, maintenance of stable intracellular UMOD upon salt loading would require at least a parallel decrease in the extracellular secretion rate. In TAL, after intracellular maturation, UMOD is membrane-anchored via glycosylphosphatidylinositol and later cleaved by the serine protease hepsin into the urine [29]. A recent study in hepsin-deficient *Hpn^Hlb320/Hlb320^* mice (Hlb320) shows that hepsin-mediated processing of UMOD is important for salt-sensitivity [10]. Thus, we quantified renal hepsin in both WKY and SHRSP. Hepsin expression in WKY was significantly higher than SHRSP (FC = 1.4, *P*=0.010), however, salt-loading did not change the hepsin expression in SHRSP (Figure 4E). Our data suggest that hepsin is not salt-sensitive in our rat models and differences in UMOD excretion on salt loading are not due to changes in hepsin expression.

### Salt-loading leads to accumulation of UMOD in SHRSP kidney

**Figure 5:**
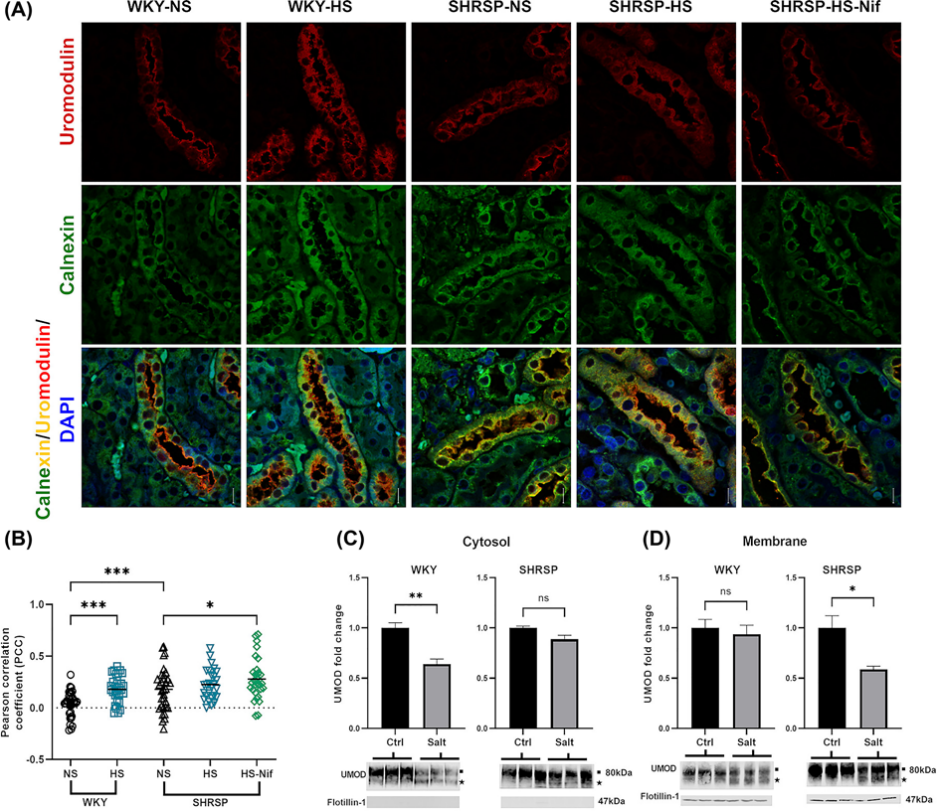
Salt loading effected the UMOD trafficking in WKY and SHRSP (**A**,**B**) displays representative immunofluorescence analysis showing UMOD (red) and ER marker calnexin (green) in rat kidney sections. Co-localization of UMOD and calnexin is represented by yellow. Nuclei are stained in blue with DAPI. Scale bar represents 20 µm. Pearson’s correlation coefficients were calculated for four representative tubules (ROI, regions of interest) from eight samples per group. One-way ANOVA was performed to determine statistical significance. (**C**,**D**) represent changes in UMOD expression of WKY and SHRSP in cytosolic and membrane fractions of TAL tubule, respectively, when incubated *ex-vivo* with mannitol (Ctrl) and NaCl (Salt). Western blots are representative images for two experiments with *n*=4 WKY and *n*=4 SHRSP. Flotillin-1 was used as membrane marker. Filled black square and filled black star on blots represent the mature UMOD and immature/precursor UMOD. Blots were normalized to total protein intensity using Revert 700 stain. Ns: non-significant, *P<0.05, **P<0.01 and ***P<0.001 (Student’s *t* test). Bars indicate mean ± s.e.m.

UMOD is a heavily glycosylated protein with a complex tertiary structure due to a high number of disulfide bonds. Therefore, processing in the endoplasmic reticulum (ER) is the rate-limiting step in UMOD maturation [30]. We hypothesized that UMOD accumulates in cellular compartments in TAL. We performed co-localization immunofluorescence analysis using calnexin as an ER marker to understand the distribution of UMOD in medullary TAL tubules of the kidney. This showed the accumulation of UMOD in the ER in SHRSP with and without salt loading (Figure 5A,B). Nifedipine treatment of salt-loaded SHRSP showed similar ER accumulation compared with normal salt and high salt SHRSP (Figure 5A,B). However, we observed increased UMOD intensity on the luminal membrane and less in ER in salt-load WKY (Figure 5A,B).

To confirm these observations, we performed *ex-vivo* incubation of freshly isolated medullary TAL tubules from WKY and SHRSP with 154 mM NaCl. UMOD expression was lower in the membrane fraction compared with the cytosolic fraction in SHRSP of TAL tubules incubated with salt and *vice versa* in the case of WKY (Figure 5C,D). Of note, we observed a second band of immature (precursor) UMOD (below the 80-kDa band) in salt incubated TAL of both the strains (Figure 5C,D). The presence of UMOD precursor band in tubule lysate after salt incubation indicates the role of salt in regulating the maturation and trafficking in UMOD secretion from TAL cells.

Notably, calnexin was highly expressed in the medullary UMOD positive-TAL tubules of SHRSP compared with WKY in our co-localization study (**S9**). Since UMOD is GPI-anchored and N-glycosylated protein, like other glycoproteins it enters the calnexin–calreticulin cycle for proper folding in ER [31]. We assessed both calnexin and calreticulin levels in kidneys and found no difference in calreticulin expression among groups (Supplementary Figure S9). However, calnexin expression in the kidney was higher in SHRSP compared with WKY and salt loading increased its expression significantly in SHRSP (*P*=0.04) (Supplementary Figure S9).

It is reasonable to infer that salt through yet unknown pathways/signaling affects the maturation and trafficking of UMOD from TAL.

## Discussion

We have recently studied total renal salt handling in response to dietary salt intake in humans [32]; our present study specially dissects the role of UMOD in the TAL in this process. We show for the first time the effect of salt as a modulator of UMOD excretion and expression against two background phenotypes: normotension (WKY) and hypertension (SHRSP). We show that salt loading decreases 24-h urinary UMOD secretion and leads to ER retention of UMOD in the chronic hypertensive model, thus affecting the molecular trafficking of UMOD secretion. Collectively, our results indicate that the difference in salt-handling and adaption to renal injury during salt stress between normotensive and hypertensive rats reflects in UMOD excretion.

In our study, irrespective of the difference in BP, both models responded to salt-loading by reduction in urinary UMOD. Studies have shown that higher salt intake leads to increased reabsorption of salt in TAL via NKCC2 activity and that UMOD plays an important role in enhancing NKCC2 activity [4,33]. Under the circumstances of high salt, it would be rational for TAL cells to reduce UMOD secretion in an attempt to reduce excess sodium reabsorption and thus playing a protective role. This hypothesis seems to be supported by the fact that in the absence of UMOD in *UMOD*^*−/*−^ mice, there is an augmented sodium excretion [5]. Our study shows that the BP component can be dissected from the salt and UMOD interaction. This is evident when the salt-induced rise in BP in SHRSP was mitigated by nifedipine treatment, while this did not restore the urinary UMOD levels to baseline. Similarly, we observed a salt-induced reduction in urinary UMOD also in salt-loaded WKY, where there is no increase in BP.

Research on salt and UMOD interaction has focused on mice and limited studies are available in rat models. A previous study by Ying et al. in kidneys of male Sprague–Dawley (SD) rats has shown that 8% dietary salt increased mRNA and protein levels of UMOD, however, urinary levels of UMOD were not investigated [34]. Contrary to this study, our data showed UMOD was unaffected at the mRNA level on 1% salt loading. The UMOD protein measurements in Ying et al.’s study [34], were obtained by membrane preparations from the medulla of SD rats, while in our study we have considered whole kidney protein levels with total intracellular (membrane and cytosolic fractions) UMOD protein level.

High dietary salt intake increases vascular oxidative stress and accelerates renal damage via increased inflammation in kidney [35–37]. Overall, there is a difference in renal adaption to salt stress in WKY and SHRSP that is reflected in UMOD maturation, folding, and trafficking. Defective trafficking and ER retention of mutant *UMOD* is known to be a key step in the pathogenesis of uromodulin-associated kidney diseases (UAKD) [30,38]. Further, ER stress induced by UMOD ER retention is known to play a role in the pathogenesis of these *UMOD*-related autosomal dominant tubulointerstitial kidney disease (ADTKD-*UMOD*) [39]. It is reasonable to hypothesize that salt induced ER stress and accumulation of UMOD in ER could lead to renal tubular cell death eventually causing kidney dysfunction and resulting in progressive renal failure. ER stress is a known contributor in pathophysiology of hypertension [40,41]. Renal ER stress induced by salt supplementation is well studied in animal models [42–45], and so is the role of oxidative stress, ER stress, and inflammation in the pathogenesis of vascular dysfunction in SHRSP [46–48]. An increase in proteinuria and NGAL, together with an increase in BP and salt supplementation, have shown to induce ER stress in the kidney [43,49].

Our study has a few limitations which should be considered while interpreting the results. We have supplemented the rats with 1% NaCl for 3 weeks, and we do not know whether the UMOD decreasing effect of salt in these models will continue past 3 weeks of salt-loading or if the intake of salt is increased. Further studies are clearly warranted to understand whether the consequences of long-term lower urinary UMOD levels (in response to salt) might have detrimental effects on renal function. In our model, systemic and/or renal ER stress might be primary or secondary to high BP, however this remains elusive.

In conclusion, we have demonstrated that in a hypertensive rat model salt decreases the secretion of UMOD and increases its ER retention and thereby demonstrated the role of salt as a modulator of UMOD expression and trafficking. Hypertensive patients harboring *UMOD* risk alleles for overexpression might be more susceptible to UMOD accumulation-induced ER stress under additional environmental challenges such as excessive salt, and this certainly warrants further study for early interventions. A better understanding of UMOD secretion, including regulation of molecular trafficking, may lead to targeted interventions with future therapeutic potential.

## Clinical perspectives

- UMOD protein is uniquely expressed in the kidneys. Although UMOD has been linked to hypertension and regulation of salt reabsorption in the tubules, the mechanisms underpinning the influence of dietary salt on UMOD excretion have not been explored.
- Herein, we show that salt-loading of both normotensive and hypertensive rat models induced a decrease in urinary UMOD excretion, with a greater effect seen in the latter. The difference in renal adaptation to salt stress was elucidated by examination of the molecular trafficking of UMOD.
- Our data raise the possibility of the design of novel therapeutics targeting molecular trafficking of UMOD.

## Supplementary Material

Supplementary Figures S1-S9

## Data Availability

All supporting data are included within the main article and supplementary data.

## Abbreviations

BP: blood pressure
CVD: cardiovascular disease
ER: endoplasmic reticulum
FC: fold change
HBSS: Hanks’ balanced saline solution
KIM-1: kidney injury marker-1
NGAL: neutrophil gelatinase-associated lipocalin
SBP: systolic blood pressure
SD: Sprague–Dawley
SHRSP: stroke-prone spontaneously hypertensive rat
TAL: thick ascending limb
UMOD: uromodulin
WKY: Wistar–Kyoto rat
