# Supplementary material for: Salt loading decreases urinary excretion and increases intracellular accumulation of uromodulin in stroke-prone spontaneously hypertensive rats
Source: Clin Sci (Lond). 2021 Dec 17;135(24):2749–61. doi: 10.1042/CS20211017 (PMC8689196; doi:10.1042/CS20211017)
Supplement: Supplementary Figures S1-S9 [file CS-2021-1017_supp.pdf]

Salt loading decreases urinary excretion and increases intracellular accumulation of uromodulin in stroke-prone spontaneously hypertensive rats.

Sheon Mary[1*], Philipp Boder[1*], Giacomo Rossitto[1, 2], Lesley Graham[1], Kayley Scott[1], Arun Flynn[1], David Kipgen[3], Delyth Graham[1], Christian Delles[1#]

[1]Institute of Cardiovascular and Medical Sciences, University of Glasgow, Scotland, UK

[2]Department of Medicine, University of Padua, Padua, Italy

[3]Department of Pathology, Queen Elizabeth University Hospital, Glasgow, UK

* Authors have contributed equally.

#Corresponding author:

Prof. Christian Delles

BHF Glasgow Cardiovascular Research Centre, Institute of Cardiovascular and Medical Sciences, University of Glasgow, 126 University Place, Glasgow G12 8TA

Email: Christian.Delles@glasgow.ac.uk

Table of content:

S1: Systolic blood pressure profile during oral salt loading.

S2: Histology data for kidney sections

S3: Baseline (11-week-old) features of rats.

S4: 24h urinary uromodulin (uUMOD) profile of each group over the period of three weeks of salt loading

S5: Correlation plot for urinary uromodulin and sodium.

S6: Expression of NKCC2

S7: Fold change of uromodulin mRNA

S8: Western blot full image and analysis for UMOD media blot

S9: Calnexin and calreticulin expression in kidney

S1: Systolic blood pressure profile during oral salt loading.

Systolic blood pressure (SBP) was monitored using the tail-cuff method. Data are shown for each group over the period of three weeks of salt loading (starting at baseline: 11-week-old rats). Each data point represents as mean ± sem of 16 rats per group and per rat 6-10 consecutive readings. WKY, Wistar Kyoto rat; SHRSP, stroke-prone spontaneously hypertensive rat; NS: normal salt; HS: high salt (1% NaCl); HS-Nif: high salt with nifedipine *Significance compared between SHRSP NS and HS, ** Significance compared between SHRSP HS and HS-Nif and # significance compared between WKY HS and SHRSP HS using two-way repeated measures ANOVA.

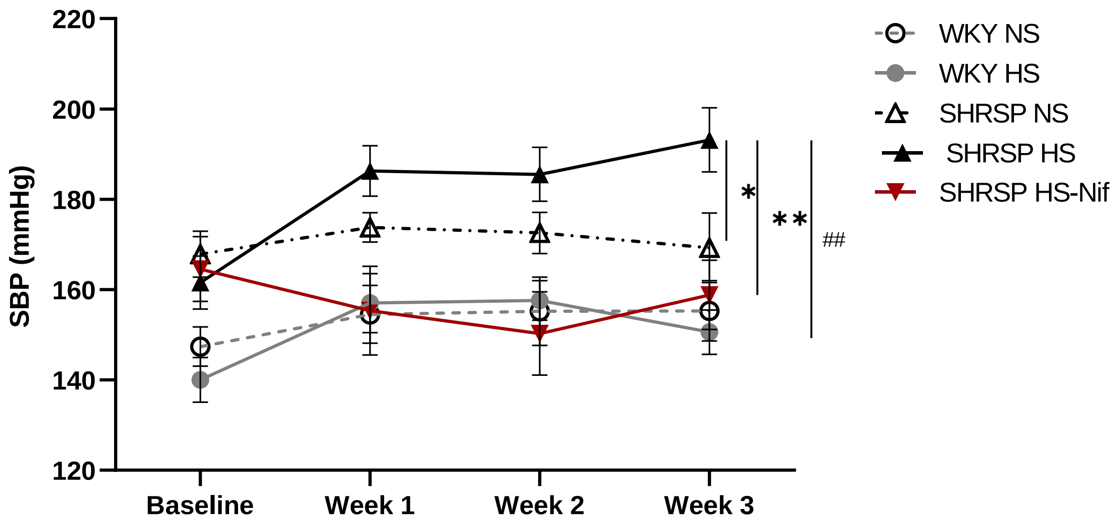

S2: Histology data for kidney sections

Panel A represents the kidney scoring criteria for glomeruli and tubules. Proximal tubules showed no acute injury in any of the groups, however, the distal tubules of SHRSP showed very mild flattening and occasional loss of tubular epithelial nuclei. n=8 per group (4 per sex) kidney sections (2µm) were evaluated and scored in a blinded fashion. Periodic acid Schiff stain was used to assess morphological differences. Graph represents the acute tubular injury. **Panel B** illustrates that male SHRSP-HS showed arteriolar hyalinosis associated with blood pressure rise in this group. Representative images of 2µm sections of kidney from each group is shown in **Panel C, D and E.** (Nif/CC-represents nifedipine)

A: g: Glomerular perfusion; 0 = glomeruli all normal, 1 = mild ischemia (moderately shrunken outer glomeruli, mild or normal inner glomeruli), 2 = severe ischemia (severely shrunken outer glomeruli, moderate or mild inner glomeruli). cv: Arterial fibrointimal thickening; 0=absent, 1=present. ah: Arteriolar Hyalinosis; 0=absent, 1=present

Sex	Animals	WKY-NS			WKY-HS			SHRSP-NS			SHRSP-HS			SHRSP-HS-Nif		
		g	cv	ah	g	cv	ah	g	cv	ah	g	cv	ah	g	cv	ah
male	1	2	0	0	1	0	0	1	0	0	2	0	1	1	0	0
male	2	2	0	0	1	0	0	1	0	0	1	0	1	1	0	0
male	3	1	0	0	1	0	0	2	0	0	0	0	1	1	0	0
male	4	1	0	0	1	0	0	1	0	0	1	0	1	1	0	0
female	5	1	0	0	1	0	0	2	0	0	1	0	0	1	0	0
female	6	1	0	0	1	0	0	0	0	0	1	0	0	2	0	0
female	7	1	0	0	1	0	0	1	0	0	1	0	0	1	0	0
female	8	1	0	0	1	0	0	1	0	0	2	0	0	1	0	0
Median		1	0	0	1	0	0	1	0	0	1	0	0.5*	1	0	0

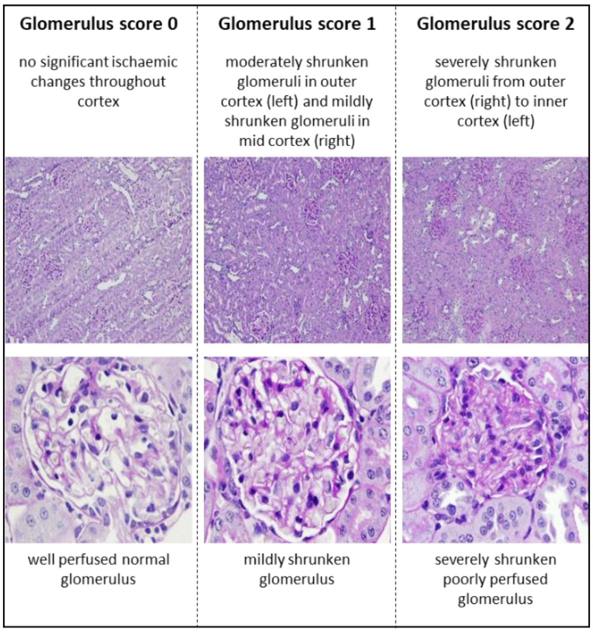

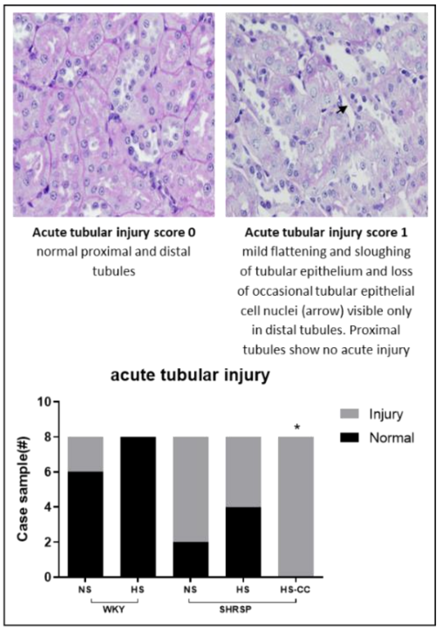

B

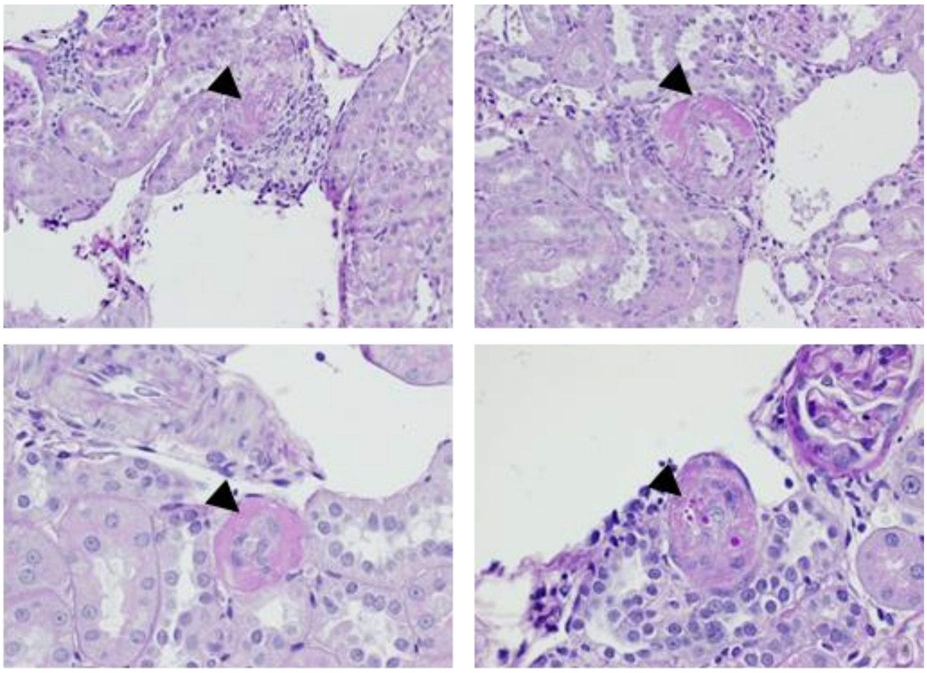

*abnormalities in arterioles (black arrow) in 4 male SHRSP-HS

C

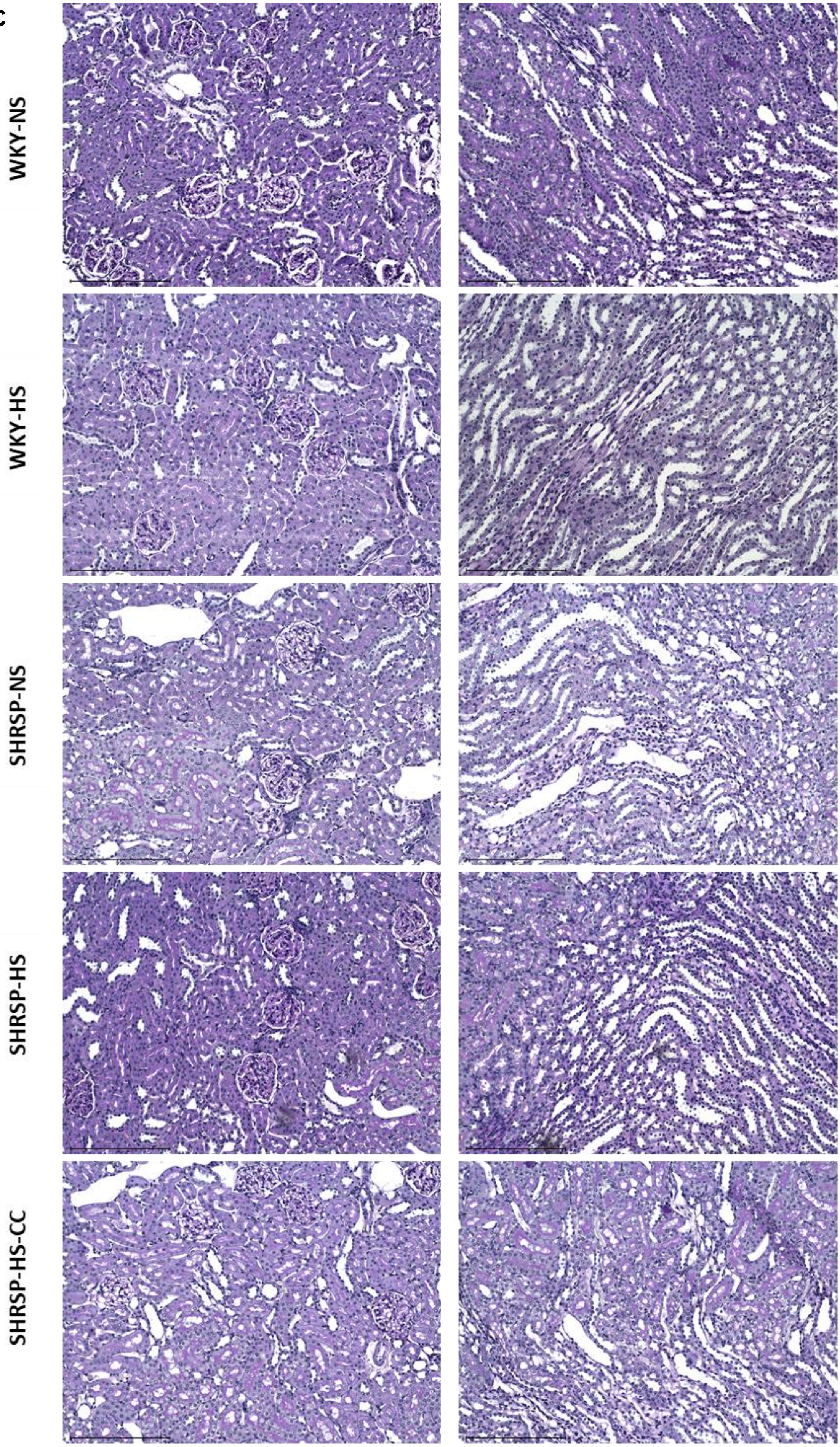

20X magnification, scale bar 200µm (bottom left corner)

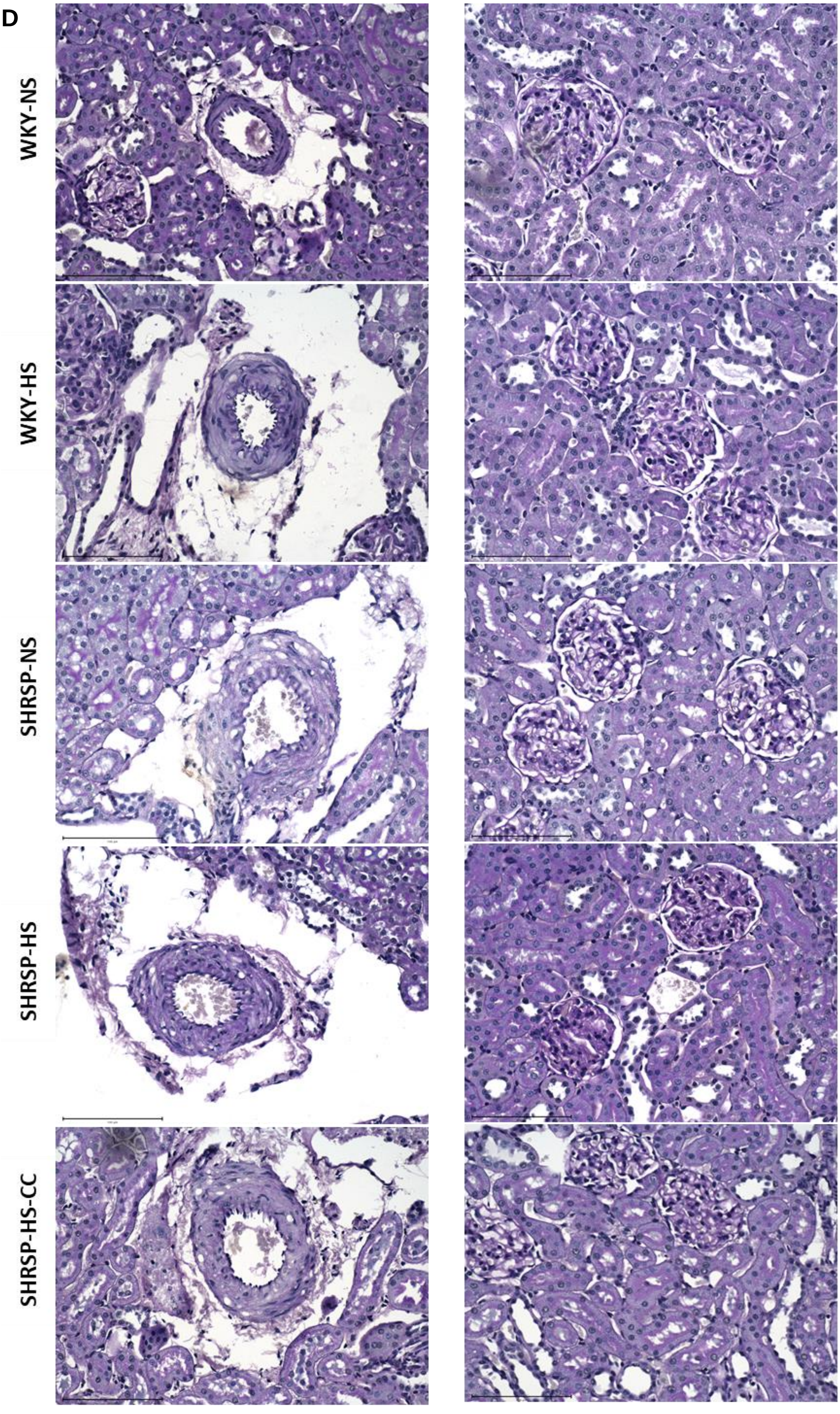

40X magnification, scale bar 100µm (bottom left corner)

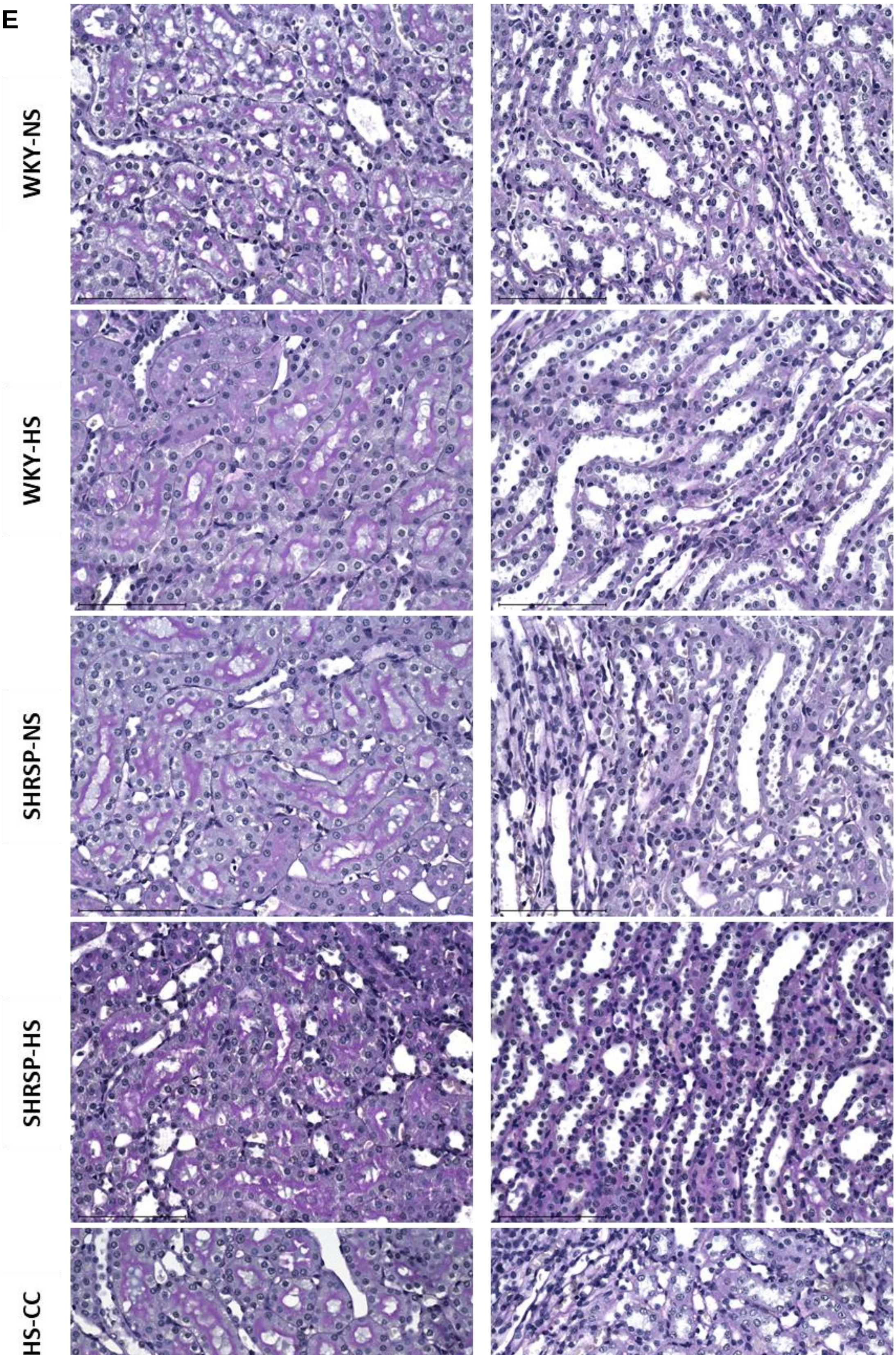

Cortex-Proximal tubule

Medullary tubules (DCT,CT, LoH)

40X magnification, scale bar 100µm (bottom left corner)

S3:

Baseline (11 week old) features of rats.

Data collected from metabolic cage study. NS: normal salt, HS: high salt (1% NaCl) and HS-Nif: high salt with nifedipine. Data represent mean ± s.e.m.

	WKY		SHRSP		
	NS	HS	NS	HS	HS-Nif
**n**	16	16	16	16	16
**Body weight (BW, g)**	203.0 ± 13.6	202.5 ± 13.9	190.7 ± 8.7	186.4 ± 8.6	193.5 ± 10.5
**Water intake (ml/h)**	1.4 ± 0.1	1.4 ± 0.1	1.2 ± 0.1	1.2 ± 0.1	1.2 ± 0.1
**Urine output (ml/h)**	0.5 ± 0.1	0.7 ± 0.1	0.4 ± 0.1	0.4 ± 0.0	0.3 ± 0.0
**u-Na^+ excretion (mmol/day)**	0.7 ± 0.1	0.9 ± 0.1	0.9 ± 0.2	0.8 ± 0.1	0.7 ± 0.1

Renal parameters after three weeks of salt-loading

Excess Na^+ balance is calculated for groups treated with 1%NaCl in drinking water as: Na^+ from solid food (estimated by average 24h Na^+ urinary excretion from NS animals) + Na^+ in drinking water - urinary Na^+ excretion; results were compared to 0 by Wilcoxon Signed Rank test, under the null hypothesis of a net Na^+ balance.

Parameters	WKY		SHRSP			2-way ANOVA	
	NS	HS	NS	HS	HS-Nif	Strain	HS
**n**	16	16	16	16	16		
**Body weight (BW, g)**	261.6 ± 13.2	267.2 ± 13.6	222.5 ± 10.1$^{\$}$	223.3 ± 10.3	225.8 ± 11.0	***	ns
**Water intake (ml/h)**	1.28 ± 0.11	2.61 ± 0.30***	1.45 ± 0.12	2.78 ± 0.19***	1.63 ± 0.10$^{\#\#\#}$	ns	***
**Urine output (ml/h)**	0.56 ± 0.06	1.80 ± 0.22***	0.53 ± 0.06	1.57 ± 0.13***	1.01 ± 0.10$^{***,\#\#}$	ns	***
**Water intake-urine output (ml/h)**	0.72 ± 0.07	0.82 ± 0.13	0.91 ± 0.09	1.21 ± 0.11*	0.63 ± 0.08$^{*,\#\#\#}$	**	0.05
**p-Na^+ (mmol/L)**	135.1 ± 1.27	136.7 ± 0.90	136.3 ± 1.04	136.0 ± 1.59	137.5 ± 0.98	ns	ns
**u-Na^+ excretion (mmol/day)**	0.83 ± 0.13	10.86 ± 1.18***	0.71 ± 0.10	10.64 ± 0.79***	7.28 ± 0.54$^{***,\#\#}$	ns	***

**Excess Na^+ balance (mmol/day)**	-	0.27 (-0.65 to 2.51)	-	1.60 (0.74 to 2.50)†	0.13 (-1.04 to 0.31) #	-	-
**p-creatinine (mmol/L)**	26.44 ± 1.39	25.25 ± 1.59	18.47 ± 1.21 $$$	20.07 ± 1.26	20.13 ± 1.24	***	ns
**u-creatinine (μmol/day)**	73.39 ± 4.72	84.41 ± 3.89*	59.37 ± 3.54 $	67.65 ± 3.40	64.51 ± 2.42	**	*

* p<0.05 vs same strain NS; $ p<0.05 SHRSP-NS vs WKY-NS; # p<0.05 vs SHRSP-HS. * p<0.05, ** p<0.01, *** p<0.001 for group comparison and 2-way ANOVA results; same for $ and #. † ≠ 0, p<0.05. NS: normal salt, HS: high salt (1% NaCl) and HS-Nif: high salt with nifedipine. Data are presented as mean ± sem

S4: 24h urinary uromodulin (uUMOD) profile of each group over the period of three weeks of salt loading (starting at baseline:11-week-old rats)

uUMOD was measured with ELISA and data point represent mean ± sem of 16 rats per group. NS: normal salt; HS: high salt (1% NaCl); HS-Nif: high salt with nifedipine. *Significance compared with NS of specific strain at a given time point using mixed model ANOVA with repeated measures.

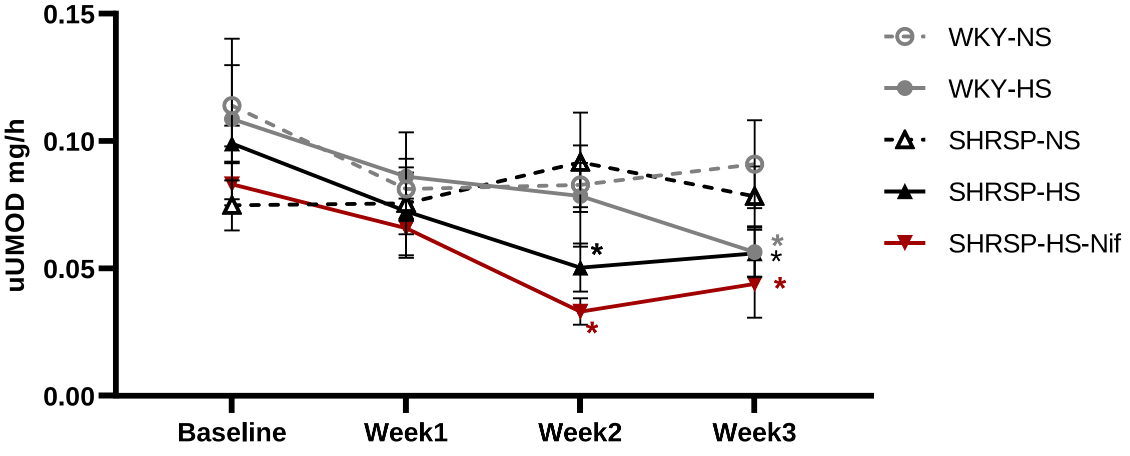

S5: Correlation plot for urinary uromodulin and sodium.

Spearman correlation showing inverse association of urinary uromodulin and urinary (r=-0.502, p=0.0005) and fractional (r=-0.368, p=0.016) excretion of sodium upon salt-loading (irrespective of strain or group). NS: normal salt, HS: high salt (1% NaCl).

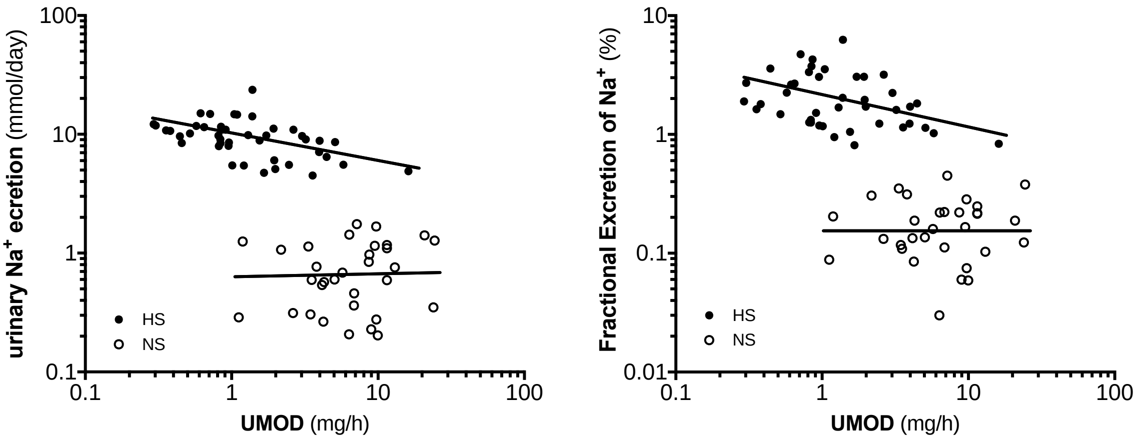

S6: Expression of NKCC2

There was no statistically significant difference in mRNA and protein expression levels of NKCC2 between groups. The sample used are kidney lysate from n=9 (protein) and n=18 (mRNA) from each group NS: normal salt; HS: high salt (1% NaCl); HS-Nif: high salt with nifedipine. Symbols with grey fill indicates female.

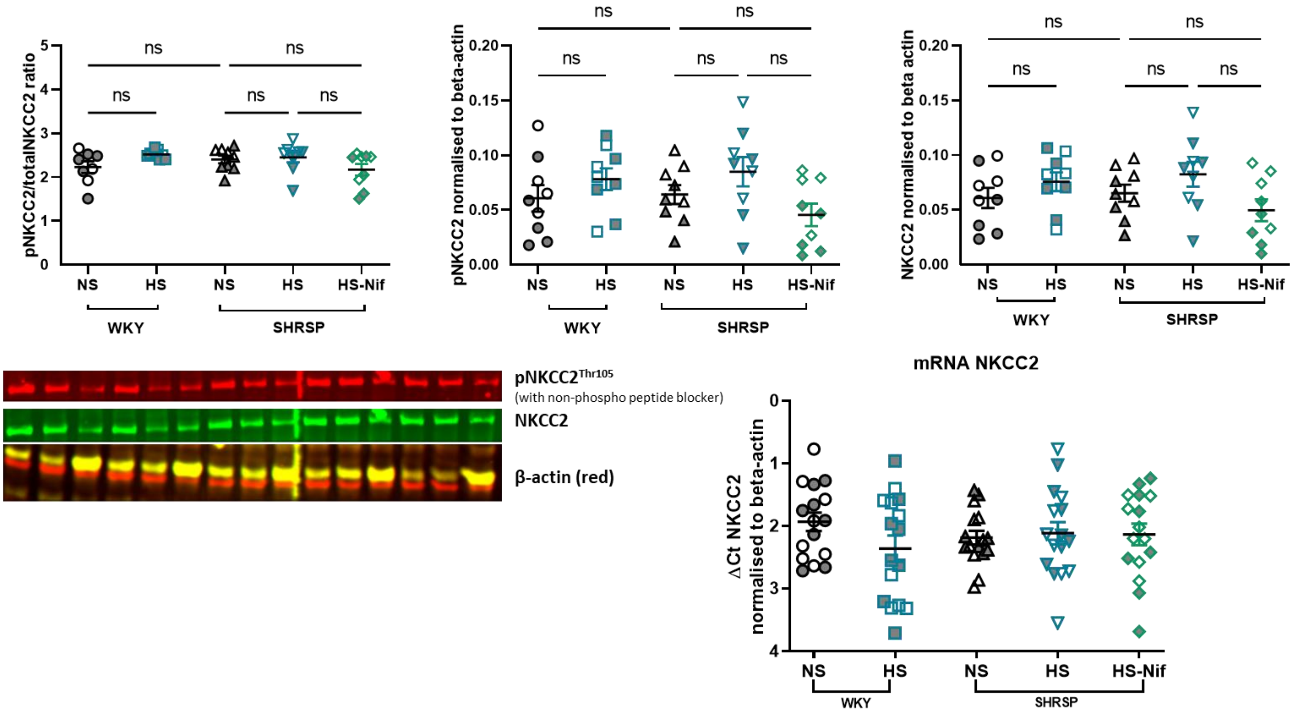

S7: Fold change of uromodulin mRNA

There was no statistically significant difference between fold change or 2^-ddCt of uromodulin mRNA across groups. NS: normal salt; HS: high salt (1% NaCl); HS-Nif: high salt with nifedipine, n=16 per group.

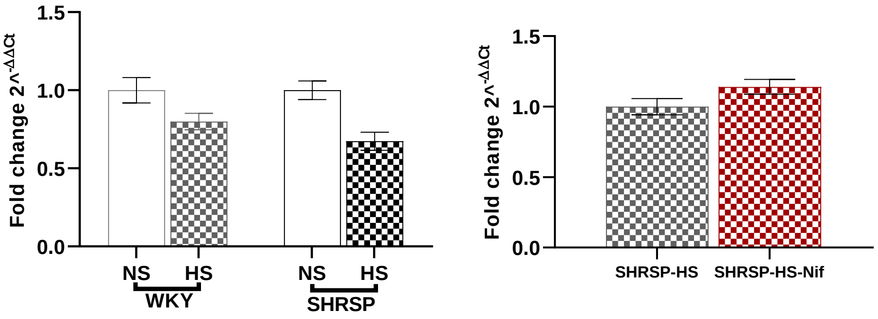

S8: Western blot full image and analysis for UMOD media blot

In *ex vivo* tubule experiments, the Western blot were performed by multi-strip blotting technique. In summary the samples run in different gels were all cut (based on molecular weight) and blotted/transferred onto a single PVDF membrane and later blocked and incubated with antibodies. Fluorescence detection was done using Odyssey Clx. We also had Western blot for recombinant uromodulin standards in varying concentration to plot standard curve. This was used to compare the intensity and estimate the amount of uromodulin in the sample. This technique of analysis was used because of absence of housekeeping/ normalizer protein in the media of *ex vivo* culture.

Extracellular media blot:

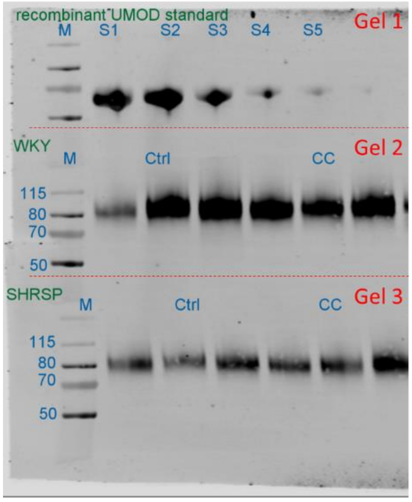

Cell lysate blot:

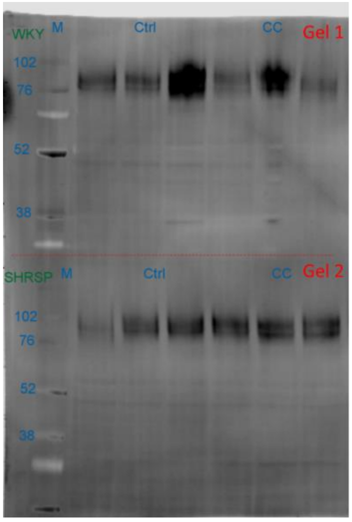

S9: Calnexin and calreticulin expression in kidney

Representative immunofluorescence images showing increased ER calnexin (green) in TAL tubules (UMOD-red) in salt-loaded rat kidney sections. n=8 tubules per kidney sections of 4 rats per group were analysed. Scale bar represents 50µm. Calnexin expression was observed to increased in UMOD-positive medullary TAL tubules in SHRSP. This was also confirmed with Western blot for calnexin in kidney lysate. There was no statistically significant difference in calreticulin expression between groups. Blots are representative images for each group. Symbols with grey fill indicates female. *p<0.05, ** p<0.01, *** p<0.001 (Brown-Forsythe Welch ANOVA test). Symbols with grey fill indicates female. Bar indicate mean ± s.e.m. n=8 per group. NS: normal salt, HS: high salt (1% NaCl) and HS-Nif: high salt with nifedipine.

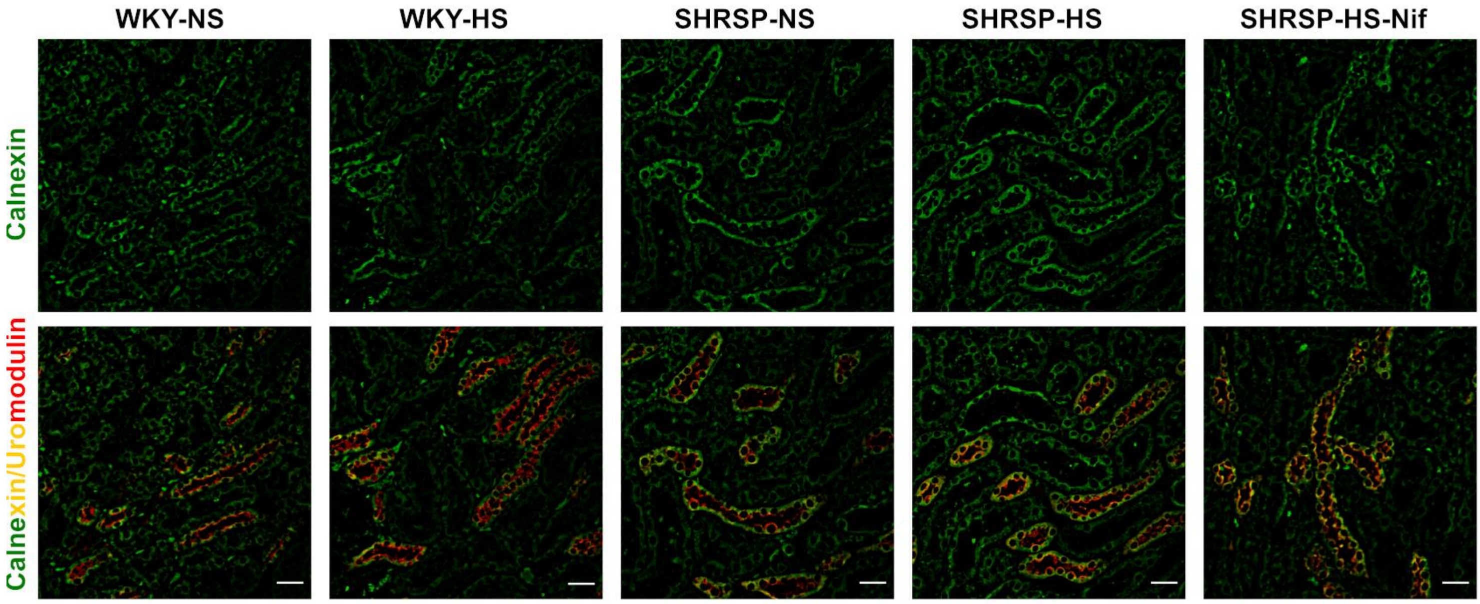

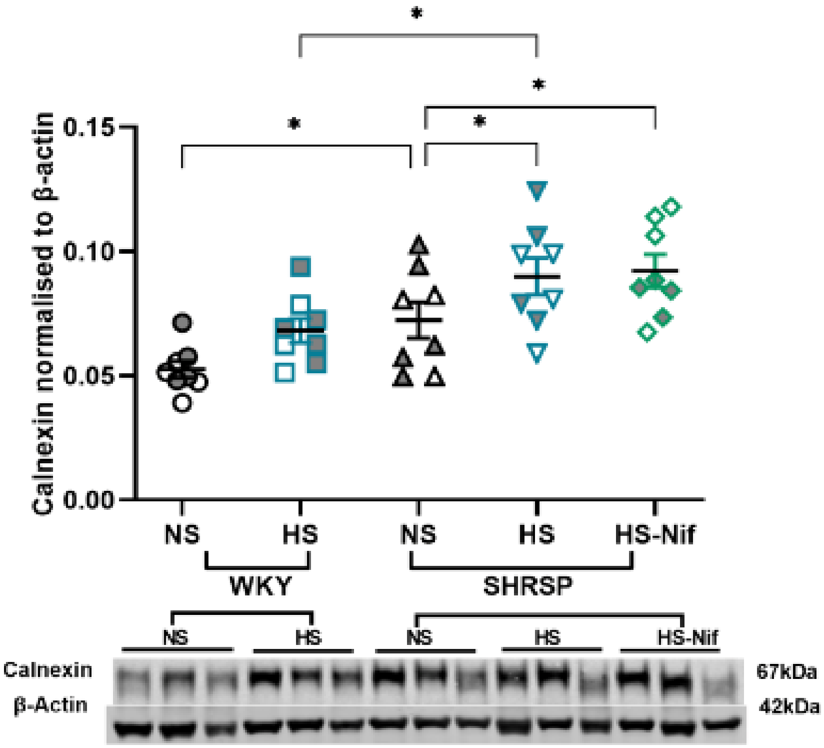

Calnexin normalised to β-actin
0.15
0.10
0.05
0.00
*
*
*
*
NS
HS
NS
HS
HS-Nif
WKY
SHRSP
NS
HS
NS
HS
HS-Nif
Calnexin
β-Actin
67kDa
42kDa

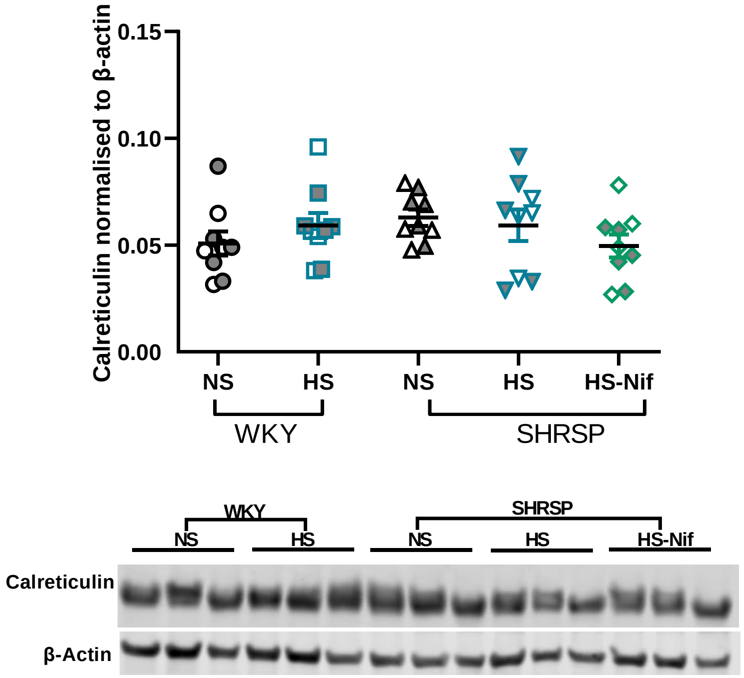

Calreticulin normalised to β-actin
0.15
0.10
0.05
0.00
NS
HS
NS
HS
HS-Nif
WKY
SHRSP
WKY
SHRSP
NS
HS
NS
HS
HS-Nif
Calreticulin
β-Actin
